# Tuning Supramolecular Structure in Trimethylglycine Cocrystals: Competition Between Hydrogen and Halogen Bonding upon Cl/Br Substitution

**DOI:** 10.3390/molecules31061047

**Published:** 2026-03-21

**Authors:** Andrei V. Churakov, Alexander G. Medvedev, Anastasia V. Shishkina, Nikita E. Frolov, Mikhail V. Vener

**Affiliations:** 1 N. S. Kurnakov Institute of General and Inorganic Chemistry, Russian Academy of Sciences, Leninskii Prosp. 31, 119991 Moscow, Russia; churakov@igic.ras.ru (A.V.C.); medvedev.chem@gmail.com (A.G.M.); 2 Scientific Department, Northern (Arctic) Federal University, Severnaya Dvina Emb. 17, 163001 Arkhangelsk, Russia; shishkina.av@gmail.com; 3 V. M. Gorbatov Federal Research Center for Food Systems, Talalikhina St. 26, 109316 Moscow, Russia; frolovne24@gmail.com

**Keywords:** 2,6-dichlorophenol and 2,6-dibromophenol, dimer vs. catemeric motif, Br∙∙∙O interactions, periodic (solid-state) DFT computations, intermolecular H∙∙∙H contacts, Hirshfeld Surface Analysis vs. Bader Analysis

## Abstract

Two novel cocrystals of zwitterionic trimethylglycine (TMG) with 2,6-dichlorophenol [TMG•2,6-dichlorophenol] (1:1) and 2,6-dibromophenol [TMG•2,6-dibromophenol] (1:2) are synthesized and structurally characterized using single crystal X-ray diffraction. To estimate the energy of various intermolecular interactions, periodic DFT calculations were performed followed by Bader analysis of the crystalline electron density. TMG molecules form dimers in [TMG•2,6-dichlorophenol] (1:1). Its supramolecular structure is governed by the primary charge-assisted H-bonds (~60 kJ/mol) and supported by C–H∙∙∙O contacts (~12 kJ/mol). Cl/Br substitution introduces a more potent halogen-bonding donor. The Br∙∙∙O^−^ interaction (~10 kJ/mol) is strong enough to reorganize the packing into a catemeric motif. As a result, TMG molecules form infinite chains in [TMG•2,6-dibromophenol] (1:2). This illustrates that “fine tuning” is not merely about changing distances, but about shifting the entire energy hierarchy of the crystal. Two-dimensional fingerprint diagrams (2D diagrams) obtained from the Hirshfeld surface and Bader’s analysis of the crystalline electron density give significantly different values of the contributions of the H∙∙∙H contacts, 28% vs. 5% respectively. The main reason for this discrepancy is the large number of relatively short intermolecular H∙∙∙H contacts without a critical bond point in trimethylglycine cocrystals.

## 1. Introduction

Trimethylglycine (TMG, betaine) is widely used as a component of dietary supplements, pharmaceuticals [1,2] and cosmetics [3]. TMG plays crucial roles as an osmoprotectant and a methyl group donor, impacting various biological processes [4,5]. Recently, TMG cocrystals have become the subject of intensive experimental and theoretical studies [5,6,7,8,9,10]. This compound is zwitterionic in both gas and condensed phases. Betaine carboxylate group forms intermolecular hydrogen bonds with the OH group(s) of coformer molecules thereby playing a pivotal role in directing the supramolecular architecture of multicomponent TMG crystals [11]. In the context of crystal engineering, the precise tuning of supramolecular structures is paramount. In the present study, 2,6-dibrimophenol [12,13] and 2,6-dichlorophenol [14] were chosen as coformer molecules. They represent very convenient objects for studying the effect of Cl/Br substitution on the supramolecular organization in TMG crystals, since this substitution hardly changes the geometric parameters of the dihalogenated phenol. Recent work directly demonstrates how single-atom halogen substitution (Cl/Br) can modulate the balance between hydrogen bonds, halogen bonds, and π–π interactions, thereby fine-tuning supramolecular architecture [15]. The interactions of Hal∙∙∙O, Hal∙∙∙H, Hal∙∙∙Hal, etc., in organic crystals, where Hal = Cl and Br, have been intensively studied recently, e.g., see [15,16,17,18,19,20], particularly regarding the competition between hydrogen and halogen bonding motifs [21,22,23] and the distinct yet complementary role of halogen bonds in crystal engineering [24]. However, the specific influence of Cl/Br substitution on the nature of secondary interactions and, consequently, on the overall supramolecular organization in organic crystals, has received comparatively less attention [25,26]. Note that in most cases, Cl/Br substitution slightly affects the supramolecular architecture of the studied crystals [27,28,29,30].

In addition to intermolecular hydrogen and halogen bonds, a large number of different non-covalent (intermolecular) interactions are realized in organic crystals [31,32,33,34,35,36]. Multicomponent systems often exploit bifunctional halogen/hydrogen bond donors to engineer complex architectures [37], including quaternary cocrystals assembled through synergistic halogen and hydrogen bonding [38], while halogen bonding can also compete with alternative reaction pathways during co-crystallization [39].

These interactions are commonly analyzed using the Hirshfeld surface calculated by CrystalExplorer [40,41]. A prevalent approach involves constructing two-dimensional (2D) fingerprint diagrams to quantify the contribution of each interaction type to the overall surface area [42,43,44]. In many organic and pharmaceutical multicomponent crystals, 2D fingerprint diagrams frequently indicate a substantial contribution from H∙∙∙H contacts, often ranging from 20% to 50% [8,42,43,44,45,46,47,48,49]. Nevertheless, two critical aspects have often been overlooked in these studies: the verification of the reported H∙∙∙H contact contributions and a thorough discussion of their structure-directing role.

**Scheme 1:**
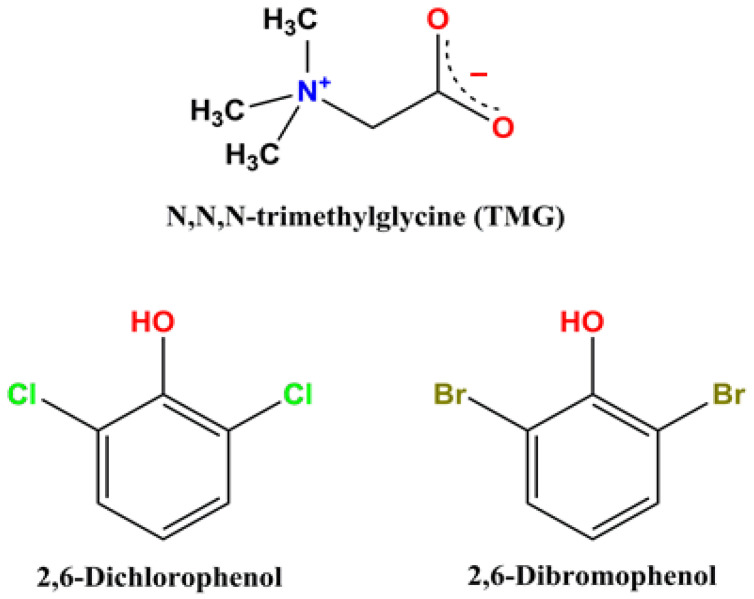
Molecular structures of TMG, 2,6-dichlorophenol and 2,6-dibromophenol.

This study aims to address these gaps by synthesizing and structurally characterizing two multicomponent crystals of TMG with 2,6-dichlorophenol and 2,6-dibromophenol (Scheme 1). By employing periodic (solid-state) DFT calculations followed by the Bader analysis of the crystalline electronic density [50], we will identify and quantify the intermolecular interactions within these crystals. This comprehensive data will enable a direct comparison of the H∙∙∙H contact contributions to the total energy of intermolecular interactions, as derived from Bader analysis, with those obtained from 2D fingerprint diagrams. A comparison of our results with literature data underscores the importance of interpreting 2D fingerprint plots cautiously when examining close contacts derived from Hirshfeld surfaces. We advocate that, in addition to standard CrystalExplorer analysis [51], periodic [47] or non-periodic [52] DFT calculations are essential for obtaining reliable estimates of H···H contributions to the total energy of intermolecular interactions in two-component organic crystals, including those of pharmaceutical relevance.

## 2. Results

### 2.1. Crystal Structures

In the present work, two crystals [TMG•2,6-dichlorophenol] (1:1) and [TMG•2,6-dibromophenol] (1:2), were obtained and structurally characterized (Table S1).

**Figure 1:**
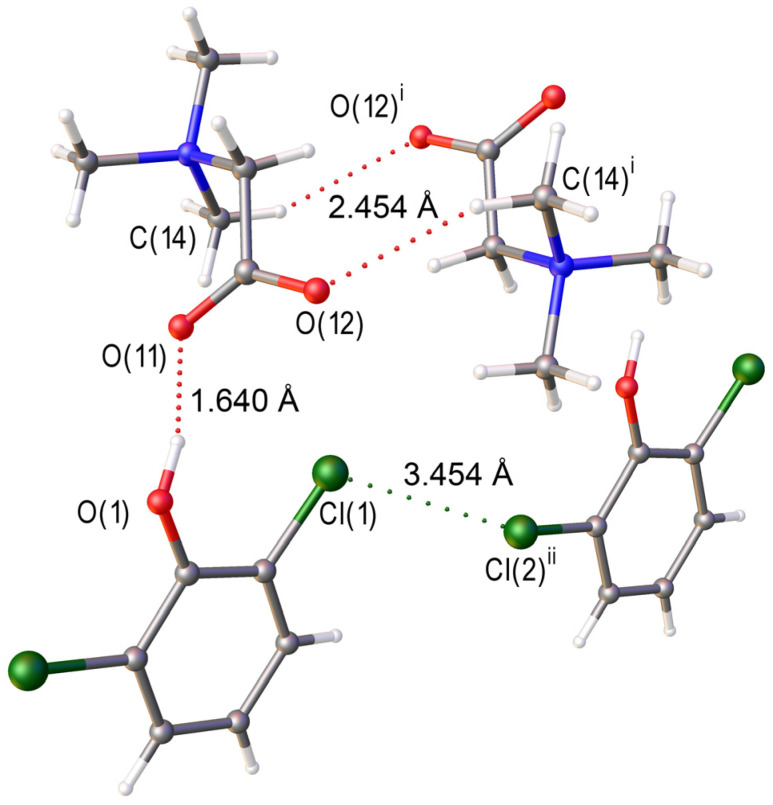
Fragment of crystal structure [TMG•2,6-dichlorophenol] (1:1). Hydrogen bonds and other intermolecular interactions are drawn as dashed lines. Symmetry operations: (i) 2 − x, 1 − y, 2 − z; (ii) 1 + x, +y, +z.

A fragment of the crystal structure of [TMG•2,6-dichlorophenol] (1:1) is shown in Figure 1. The asymmetric unit of the [TMG•2,6-dichlorophenol] structure comprises one independent TMG molecule and one nearly planar 2,6-dichlorophenol molecule (Figure S1). The metric parameters of both components are consistent with those reported for their single-component crystalline forms [53]. In the phenol derivative, the C(1)–O(1)–H angle is 118(2)°. A 1:1 heterosynthon is formed via a charge-assisted hydrogen bond O(1)–H∙∙∙O(11), with a short donor-acceptor distance O(1)∙∙∙O(11) of 2.512(1) Å. It is noteworthy that the second oxygen atom of the carboxyl group does not engage in hydrogen bonding. In addition to O(1)–H∙∙∙O(11) bonds, the crystal structure exhibits Cl(1)∙∙∙Cl(2) contacts with a separation of 3.454(1) Å (Table 1). Further identification and quantitative assessment of the halogen bond energy require periodic DFT calculations.

**Figure 2:**
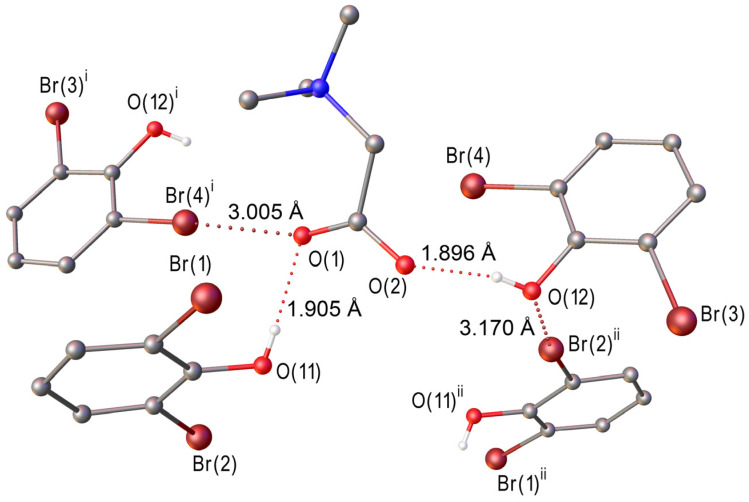
Fragment of crystal structure [TMG•2,6-dibromophenol] (1:2). Hydrogen bonds and other intermolecular interactions are drawn as dashed lines. Hydrogen atoms of alkyl and aryl groups of TMG are omitted for clarity. Symmetry operations: (i) 1 − x, 1 − y, 1 − z; (ii) 1 − x, 1 − y, 2 − z.

Replacing the chlorine atoms in 2,6-dichlorophenol with bromine induces significant changes in the hydrogen bonding network and non-covalent interactions (cf. Figure 1 with Figure 2). Unlike the 1:1 heterosynthon in [TMG·2,6-dichlorophenol], the structure of [TMG·2,6-dibromophenol] comprises one independent TMG zwitterion and two crystallographically independent 2,6-dibromophenol molecules (Figure S2). In this case, both carboxyl oxygen atoms of TMG participate in charge-assisted hydrogen bonds, forming a 1:2 heterosynthon with an O(11)∙∙∙O(1) distance of 2.624(2) Å and an O(12)∙∙∙O(2) distance of 2.617(2) Å. Notably, the refined O–H distance in the 2,6-dibromophenol molecules is 0.74(3) Å, significantly shorter than that observed in the chlorinated analogue (0.90(2) Å). The crystal packing features two distinct halogen bonds: a Br(2)∙∙∙O(12) contact of 3.170(1) Å between adjacent phenol derivatives and Br(4)∙∙∙O(1) interaction of 3.005(1) Å linking TMG and 2,6-dibromophenol molecules (Figure 2). The significantly shorter O–H distance in 2,6-dibromophenol may be attributed to the presence of halogen bonding interactions (Table 1).

Molecular Electrostatic Potential (ESP) surface analysis was used to visualize regions of electron excess and deficiency in halogenated phenol molecules, supporting the possible formation of Hal···O halogen bonds. (Detailed information on ESP calculations is provided in the Supplementary Materials). For comparison, both chlorinated and brominated analogues were analyzed (Figure S3). According to calculations, the ESP maxima values at Br atoms in 2,6-dibromophenol (86.2 and 51.3 kJ/mol) are higher than corresponding values at Cl atoms in 2,6-dichlorophenol (64.5 and 27.8 kJ/mol). The obtained values clearly indicate the greater propensity of the brominated analogue to form Hal···O halogen bonds compared to the chlorinated analogue.

### 2.2. Periodic DFT Calculations Followed by Bader Analysis of the Crystalline Electron Density

This study proposes that a specific non-covalent intermolecular interaction is associated with the existence of a bond path (i.e., a bond critical point) between a pair of atoms [54]. The absence of a bond critical point means that the two atoms do not immediately interact. The electron density, the Laplacian of the electron density at the bond critical point and the energy *E*_int_ of the strongest H-bonds and non-covalent interactions of different types in [TMG•2,6-dichlorophenol] (1:1) and [TMG•2,6-dibromophenol] (1:2) are given in Table 2 and Table S2, respectively. It follows that all these interactions meet the criteria for closed-shell attractions [50].

The energy of charge-assisted hydrogen bonds O–H∙∙∙O varies from ~60 kJ/mol in [TMG•2,6-dichlorophenol] (1:1) to ~40 kJ/mol in [TMG•2,6-dibromophenol] (1:2) (Table 2 and Table S2). A strong hydrogen bond [55] in the first crystal is caused by a presence of two chlorine atoms in orto-position to the OH group in the phenol moiety [56,57]. According to the literature [58,59], PBE-D3 calculations overestimate the energy of moderate and strong hydrogen bonds compared to B3LYP calculations (Table 2 and Table S2). This is because PBE-D3 underestimates the distance between heavy atoms that form a hydrogen bond (Table 1 from this paper and Table S7 in [58]). It should be noted that the dispersion correction does not affect this conclusion [60].

**Figure 3:**
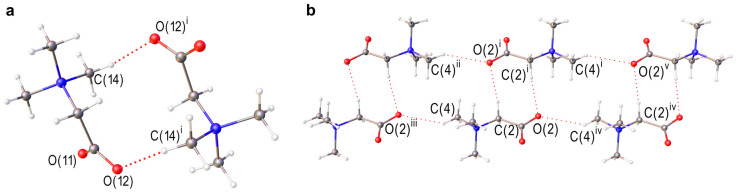
Supramolecular organization of TMG molecules in the studied crystals: (**a**) dimers in [TMG•2,6-dichlorophenol] (1:1) and (**b**) infinite chains in [TMG•2,6-dibromophenol] (1:2), C–H∙∙∙^−^O bonds are drawn as dashed lines. Symmetry elements: frame (**a**) (i) 2 − *x*, 1 − *y*, 2 − *z*; frame (**b**) (i) 2 − *x*, 1 − *y*, 1 − *z*; (ii) 2 − *x*, 1 − *y*, −*z*; (iii) +*x*,+*y*, −1 + *z*; (iv) +, +*y*, 1 + *z*; (v) 2 − *x*, 1 − *y*, 2 − *z*.

The crystals under consideration also contain C–H∙∙∙O bonds. They are realized between TMG molecules (Figure 3 and Table 1) and their energy is around ~12 kJ/mol in [TMG•2,6-dichlorophenol] (1:1) and ~9 kJ/mol in [TMG•2,6-dibromophenol] (1:2). The obtained energy values are consistent with the literature data [9,61,62,63]. TMG molecules form dimers in [TMG•2,6-dichlorophenol] (1:1) and infinite chains parallel to *c* axis in [TMG•2,6-dibromophenol] (1:2); see Figure 3. To explain the change in the supramolecular structure caused by the replacement of chlorine with bromine, it is necessary to take into account the number and energy of the intermolecular interactions formed by the oxygen atoms of the carboxylate group.

In [TMG•2,6-dichlorophenol] (1:1), each carboxylate group forms one O–H∙∙∙O and one C–H∙∙∙O bond (Figure 1). The energy of Cl∙∙∙O interaction is less than 8 kJ/mol and is significantly lower than the energy of C–H∙∙∙O bonds (Table 2). In [TMG•2,6-dibromophenol] (1:2), each carboxylate group participates in the formation of two O–H∙∙∙O bonds and one Br∙∙∙O interaction (Figure 2), the energy of which is about 10 kJ/mol. This is the strongest interaction after the O–H∙∙∙O bond (Table S2). Thus, the change in the supramolecular structure of the studied crystals upon Cl/Br substitution is due to a change in the type of the strongest secondary interactions.

A large number of diverse non-covalent interactions with energies below 7 kJ/mol are realized in the crystals (Table 2 and Table S2). The main contribution to the energy of intermolecular interactions is made by hydrogen bonds, while the contribution of H···H contacts does not exceed 10% (Tables S3 and S4). This is consistent with the results obtained previously for multicomponent drug crystals [32,42,44,47,64], hydrates of drug-like molecules [65,66] and peroxosolvates of (Nitropyrazolyl)furazans [58]. On the other hand, 2D fingerprint diagrams show a much larger contribution of H∙∙∙H contacts for two-component organic crystals, including pharmaceutical multicomponent crystals [8,42,43,44,45,46,47,48,49].

**Figure 4:**
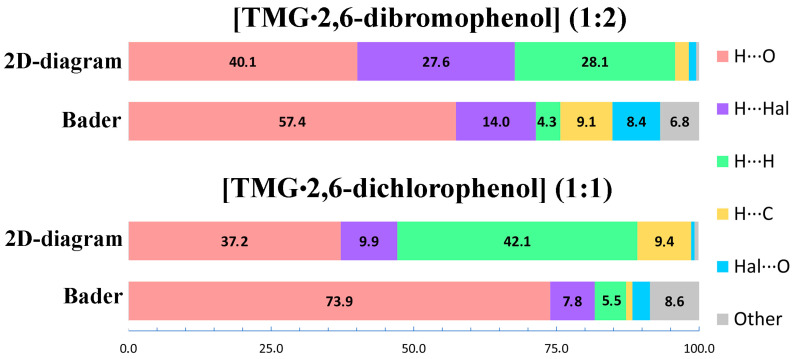
The relative contribution of intermolecular hydrogen bonds (H∙∙∙O) and various non-covalent interactions to the total energy of intermolecular interactions in the crystals. Two-dimensional fingerprint diagrams (2D diagram) vs. Bader analysis of the crystalline electron density. Hal represents Cl or Br. Numerical labels are omitted for contact contributions below 3%.

To address this issue, 2D fingerprint plots for the TMG molecule in [TMG•2,6-dichlorophenol] (1:1) and [TMG•2,6-dibromophenol] (1:2) were evaluated (Figure S4). The relative contributions of the intermolecular contacts to the Hirshfeld surface area are compared with the results obtained using Bader’s analysis of the crystalline electron density; see Figure 4. It follows that 2D diagrams almost halve the contribution of hydrogen bonds and overestimate the contribution of H∙∙∙H contacts several times. The possible reason for this discrepancy is discussed in the next section.

## 3. Discussion

The transition from TMG dimers in [TMG•2,6-dichlorophenol] (1:1) to infinite chains in [TMG•2,6-dibromophenol] (1:2) is a clear demonstration of how crystal architecture can be fine-tuned by subtle changes in intermolecular forces. In the chlorinated system, the structure is governed by the primary charge-assisted H-bonds and supported by C–H∙∙∙O contacts. However, the introduction of bromine introduces a more potent halogen-bonding donor. The Br∙∙∙O interaction (~10 kJ/mol) is strong enough to reorganize the packing into a catemeric motif. This illustrates that “fine tuning” is not merely about changing distances, but about shifting the entire energy hierarchy of the crystal. To understand this tuning, we must look beyond the primary H-bonds. While the O–H∙∙∙O bonds provide the “scaffold” (~60 kJ/mol), the secondary interactions (halogen bonds and weak C–H∙∙∙O contacts) act as the “steering forces” that determine the final architecture. Our periodic DFT calculations quantify this hierarchy, showing that even a difference of 2–3 kJ/mol in secondary interactions is sufficient to trigger a complete change in the 3D-network. The ability to fine-tune crystal structures relies on our ability to accurately describe and quantify these steering forces. Here, we encounter a significant methodological discrepancy. Standard Hirshfeld surface analysis, widely used to “summarize” interactions, suggests that H∙∙∙H contacts are a dominant factor in the stability of these TMG cocrystals (>28% contribution, Tables S3 and S4).

When comparing the Hirshfeld surface analysis and the Bader analysis, it must be taken into account that they use *different* positions of H atoms in the crystals. The Hirshfeld surface analysis is based on X-ray data. The Bader analysis uses crystalline electron density obtained from precise X-ray experiments or periodic DFT calculations; see, for example, [67]. The difference in the values of the H∙∙∙H distances obtained using the approaches under consideration can be more than 0.1 Å (Table 1). To estimate the error in determining the contribution from H...H contacts, caused by the use of different positions of hydrogen atoms, intermolecular interactions in naphthalene and anthracene crystals were calculated. According to Table 3, the Hirshfeld surface analysis and the Bader analysis give similar results. It should be noted that a large number of H∙∙∙H contacts in naphthalene and anthracene crystals represent closed-shell interactions (Tables S5 and S6). We conclude that the uncertainty in the positions of hydrogen atoms leads to differences in the H∙∙∙H contributions calculated using the two approaches of about 10% (Table 3).

It is obvious that there must be another reason for the significant difference in the contributions of the H∙∙∙H contacts obtained using the two approaches for the crystals under consideration, multicomponent drug crystals [42,44,45,46,47], etc. [58,68]. Unlike naphthalene and anthracene, these multicomponent crystals contain moderate and strong hydrogen bonds. To identify this reason, we performed calculations for a number of single-component crystals with hydrogen bonds of different types and strengths. The ideal object is ice II, since it has been studied by neutron diffraction [69]. Each hydrogen atom participates in one hydrogen bond and two short H···H contacts (<2.3 Å). However, topological analysis of the crystalline electron density reveals that only the hydrogen bond possesses a bond critical point, whereas the H···H contacts remain topologically unbound (Table 3). Similar situations have been documented for formic acid, cytosine, 3-deazauracil, N-formylglycine (Table 3, Tables S7 and S8). Despite this, Hirshfeld fingerprint plots routinely assign a substantial surface contribution to H···H contacts, often ranging from 10–40%. This discrepancy arises because the Hirshfeld approach classifies contacts purely by interatomic distances, whereas Bader analysis defines interactions through electron-density topology. As a result, many geometrically short H···H contacts represent mere spatial proximity rather than closed-shell interactions.

**Table 3:** Relative contributions of hydrogen bonds and non-covalent interactions to the total energy of intermolecular interactions in crystalline naphthalene, anthracene, water ice II and formic acid obtained using Bader analysis of the crystalline electron density ^(a)^ (Bader) and two-dimensional fingerprint diagrams (2D-diagram) [70].

Contact	Naphthalene ^(b)^		Anthracene ^(c)^	
	Contribution in %			
	Bader	2D-Diagram	Bader	2D-Diagram
C∙∙∙H	38.0	45.0	39.0	51.0
H∙∙∙H	62.0	54.0	61.0	48.0
Other	0.0	1.0	0.0	1.0
	**Water ice II ^(d)^**		**Formic acid ^(e)^**	
H∙∙∙O	96.0	49.5	83.5	74.2
H∙∙∙H	0.0	49.9	0.0	10.3
O∙∙∙O	4.0	0.6	5.0	5.7
H∙∙∙C	-	-	0.0	6.1

^(a)^ periodic DFT computations using the PBE-D3 level; ^(b)^ CSD Ref. code is NAPHTA33; ^(c)^ CSD Ref. code is ANTCEN01; ^(d)^ database code ICSD is 15907; ^(e)^ CSD Ref. code is FORMAC01.

Our results provide a direct illustration of this general phenomenon. Hirshfeld surface analysis suggests a dominant role for H···H contacts in the packing of the studied crystal. However, Bader’s analysis shows that their energetic contribution is disproportionately small, accounting for less than 5% of the total interaction energy, and, critically, that most of these contacts lack bond paths. Thus, what appears in the Hirshfeld description as a major stabilizing factor is, in fact, energetically negligible. This apparent contradiction—significant surface overlap without corresponding electron-density bonding—highlights a fundamental limitation of distance-based descriptors. If crystal packing were interpreted solely through Hirshfeld fingerprints, the fine-tuning mechanism would be incorrectly attributed to H···H stabilization. The topological analysis instead demonstrates that structurally relevant stabilization originates from interactions supported by bond critical points. Therefore, reliable analysis of crystal engineering effects requires moving byond geometric “contacts” and employing electron-density-based descriptors that reflect the true energetic landscape of the crystal.

Thus, it can be concluded that 2D diagrams can significantly overestimate the contribution from H···H contacts in crystals with strong and moderate hydrogen bonds.

## 4. Methods

### 4.1. Materials

Anhydrous trimethylglicine (>97.0 wt.%), 2,6-dichlorophenol (>99.0 wt.%) and 2,6-dibromophenol (>98.0 wt.%) were purchased from TCI Chemicals (Tokyo, Japan). Methanol (≥99.8 wt.%, Merck, Darmstadt, Germany) was dried over 3 Å molecular sieves prior to use.

**Safety Note!** Both 2,6-dichlorophenol and 2,6-dibromophenol are toxic, potent allergens, and respiratory/ocular irritants. Methanol is also highly toxic via inhalation and skin absorption, with a risk of systemic toxicity and blindness. Consequently, all synthetic procedures should be performed in a well-ventilated fume hood using advanced personal protective equipment, including double nitrile gloves with extended cuffs, safety goggles, and laboratory coats; furthermore, respiratory protection with a half-mask is mandatory. All personnel should be specifically trained in the handling of toxic solvents and halogenated compounds. Prior to any laboratory work, a comprehensive risk assessment must be conducted to identify specific hazards and mitigation strategies. Halogenated waste was collected and disposed according to established hazardous waste protocols.

### 4.2. Synthesis

Anhydrous TMG (0.0117 g, 0.1 mmol) and 2,6-dichlorophenol (0.0163 g, 0.1 mmol) were sequentially dissolved in dry methanol (0.5 mL). The solvent was evaporated to half its volume under reduced pressure using a rotary evaporator. The formation of transparent crystals was observed. The concentrated solution was then stored in a refrigerator at 4 °C overnight to ensure more complete crystallization of the adduct. Single crystals of [TMG•2,6-dichlorophenol] (1:1) were isolated from the reaction mixture and subjected to X-ray structural analysis without further purification.

Anhydrous TMG (0.0117 g, 0.1 mmol) and 2,6-dibromophenol (0.0504 g, 0.2 mmol) were sequentially dissolved in dry methanol (0.5 mL). The solution was concentrated to approximately 0.15 mL under reduced pressure using a rotary evaporator. The adduct was then crystallized by storing the concentrated solution at 4 °C overnight. Single crystals of [TMG•2,6-dibromophenol] (1:2) were isolated from the reaction mixture and subjected to X-ray structural analysis without further purification.

Dichlorophenol and dibromophenol are strong allergens. We decided not to risk conducting IR spectroscopy and powder diffraction, which require working with significant quantities of substances in open air, unlike X-ray diffraction, where we worked with a single crystal. Therefore, structural characterization was limited to single-crystal X-ray diffraction analysis.

### 4.3. Single-Crystal XRD

Experimental intensifies were measured on a Bruker D8 Venture diffractometer (Bruker AXS, Karlsruhe, Germany). Absorption corrections based on measurements of equivalent reflections were applied [71]. The structures were solved by direct methods and refined using full matrix least-squares on *F*^2^ with anisotropic thermal parameters for all non-hydrogen atoms [72]. All hydrogen atoms were found with difference Fourier synthesis and refined isotropically. Experimental details are listed in Table S1. CCDC 2480902 and 2481076 contains the supplementary crystallographic data for this paper. These data can be obtained free of charge via http://www.ccdc.cam.ac.uk/structures (or from the CCDC, 12 Union Road, Cambridge CB2 1EZ, UK; Fax: +44-1223-336033; E-mail: deposit@ccdc.cam.ac.uk).

### 4.4. Computational Details

In the CRYSTAL17 calculations [73], we employed the PBE [74] and B3LYP [75,76] functionals with the 6-31G** basis set for C, H, N, O, Cl atoms and pob_TZVP_rev2 for Br atoms [77]. The sequential introduction of dispersion corrections in the B3LYP method is not unambiguous [78,79,80]. Therefore, London dispersion interactions were taken into account by introducing the D3 correction with Becke-Jones damping [81], as implemented in CRYSTAL17, only in PBE calculations. PBE-D3/6-31G** and B3LYP/6-31G** are successfully used in the calculation of various properties of one- and multicomponent organic crystals [32,42,45,46,59,64,65,82,83]. (Further computation details are given in the Supplementary Materials).

It was assumed that intermolecular hydrogen bond or non-covalent (intermolecular) interaction is realized when there is a point (3,−1) on the bond path [50] between two atoms belonging to different molecules. Bader analysis of the crystalline electron density is carried out using Topond14 [84]. The energy of intermolecular interactions *E*_int_ is evaluated according to [85] as:

*E*_int_ [kJ/mol] = 1124 *G_b_* [atomic units], (1)

where *G_b_* is the local electronic kinetic energy density at noncovalent interaction bond critical point [50]. Equation (1) does not contain basis set superposition error (BSSE).

### 4.5. 2D Fingerprint Plot

Hirshfeld surface analysis and corresponding two-dimensional fingerprint plots were generated using CrystalExplorer (version 21.5) [51]. For this study, we selected the TMG molecule of characteristic heterosynthons [TMG•2,6-dichlorophenol] (1:1) and [TMG•2,6-dibromophenol] (1:2) as objects for Hirshfeld surface generation with standard preferences.

## 5. Conclusions

Two novel cocrystals of zwitterionic trimethylglycine (TMG) with 2,6-dichlorophenol [TMG•2,6-dichlorophenol] (1:1) and 2,6-dibromophenol [TMG•2,6-dibromophenol] (1:2) are synthesized and structurally characterized. Periodic DFT calculations were performed supplemented by Bader analysis of the crystalline electron density to estimate the energy of various intermolecular interactions. In both crystals, the O–H group of dihalogenated phenol and the carboxylate group of TMG form a charge-assisted hydrogen bond O–H∙∙∙O. Its energy varies from ~40 kJ/mol in [TMG•2,6-dibromophenol] (1:2) to ~60 kJ/mol in [TMG•2,6-dichlorophenol] (1:1). The supramolecular structure of the crystals is different: TMG forms dimers in [TMG•2,6-dichlorophenol] (1:1) and infinite chains in [TMG•2,6-dibromophenol] (1:2). This structural divergence is attributed to the change in the nature of the dominant secondary interactions upon Cl/Br substitution. In [TMG•2,6-dichlorophenol] (1:1), these are C–H∙∙∙O bonds, with the energy of ~12 kJ/mol. In [TMG•2,6-dibromophenol] (1:2), these are Br∙∙∙O^−^ interactions, with the energy of ~10 kJ/mol. This illustrates that tuning supramolecular structure upon Cl/Br substitution is not merely about changing distances, but about shifting the entire energy hierarchy of the crystal. Therefore, reliable analysis of crystal engineering effects requires moving beyond geometric “contacts” and employing electron-density-based descriptors that reflect the true energetic landscape of the crystal under consideration.

Two-dimensional Hirshfeld fingerprint plots (2D diagrams) and Bader’s analysis of the crystalline electron density reveal significantly different values of the contributions of the H∙∙∙H contacts. In the crystals studied, 2D diagrams give more than 28%, while Bader’s analysis gives less than 5%. The same trend is observed for single-component crystals with hydrogen bonds of different types and strengths. According to Bader’s analysis, proton-ordered water ice II, cytosine and formic acid lack H∙∙∙H contacts, while 2D diagrams show more than 30%. The main reason for this discrepancy is the large number of relatively short H∙∙∙H intermolecular contacts without a critical bond point. Thus, two-dimensional fingerprint diagrams should be used with caution for identifying of H∙∙∙H contacts in molecular crystals with moderate and strong hydrogen bonds. The key point is that intermolecular H∙∙∙H interactions do not play a structure-forming role and have virtually no effect on the supramolecular structure of the crystals under consideration, organic and pharmaceutical multicomponent crystals with moderate and strong hydrogen bonds.

## Figures and Tables

**Table 1:** Metric parameters of hydrogen bonds and selected non-covalent interactions in the crystals under consideration. Experiment vs. periodic DFT computations at the B3LYP and PBE-D3 levels. The angles are given in brackets. Units are Å and degrees.

Fragment/Bond ^(a)^	X-ray ^(b)^	B3LYP	PBE-D3
[TMG•2,6-dichlorophenol] (1:1)			
O(1)–H∙∙∙O(11)/H∙∙∙O(11)	**2.512(1)**/1.64(2) [163(2)]	**2.517**/1.52 [162]	**2.485**/1.49 [163]
C(14)–H∙∙∙O(12)/H∙∙∙O(12)	**3.343(2)**/2.45(2) [157(1)]	**3.285**/2.25 [157]	**3.309**/2.27 [157]
Cl(1)∙∙∙O(11)	3.252(1)	3.268	3.231
Cl(1)∙∙∙Cl(2)	3.4543(5)	3.436	3.429
H(152)∙∙∙H(5′)	2.43	2.29	2.30
[TMG•2,6-dibromophenol] (1:2)			
O(11)–H∙∙∙O(1)	**2.624(2)**/1.90(3) [166(3)]	**2.609**/1.61 [168]	**2.586**/1.57 [167]
O(12)–H∙∙∙O(2)	**2.617(2)**/1.90(3) [163(3)]	**2.618**/1.67 [158]	**2.597**/1.64 [156]
C(2)–H∙∙∙O(2)/H∙∙∙O(2)	**3.419(3)**/2.48 [158]	**3.345**/2.42 [156]	**3.359**/2.42 [156]
Br(4)∙∙∙O(1)	3.005(2)	3.020	3.010
Br(2)∙∙∙Br(4)	3.9097(4)	3.903	3.899
H(32)∙∙∙H(32″)	2.34	2.36	2.36

^(a)^ Atomic numbering is borrowed from cif files; ^(b)^ the distance between heavy atoms that form a hydrogen bond is given in bold; standard errors of distances and angles are given in parentheses.

**Table 2:** The electron density *ρ*_b_, the Laplacian of the electron density ∇^2^*ρ*_b_ at the bond critical point found from Bader’s analysis of the periodic electron density calculated at the B3LYP and PBE-D3 levels and the energy *E*_int_^(a)^ of the strongest H-bonds and non-covalent interactions of different types in [TMG•2,6-dichlorophenol] (1:1).

Fragment ^(b)^	B3LYP			PBE-D3		
	*ρ*_b_ (a.u.)	∇^2^*ρ*_b_ (a.u.)	*E*_int_ (kJ/mol)	*ρ*_b_ (a.u.)	∇^2^*ρ*_b_ (a.u.)	*E*_int_ (kJ/mol)
O(1)–H∙∙∙O(11	0.07137	0.15851	59.3	0.08823	0.12948	68.4
C1(4)–H∙∙∙O(12)	0.01558	0.04464	12.6	0.01510	0.04217	11.8
Cl(1)∙∙∙O(11)	0.00843	0.03092	7.4	0.00902	0.03307	7.9
Cl(2)∙∙∙H(131)	0.00815	0.028045	6.3	0.00886	0.02983	6.8
Cl(1)∙∙∙Cl(2)	0.00741	0.02893	6.2	0.00747	0.02857	6.1
C(2)∙∙∙H(143)	0.00871	0.02598	6.1	0.00864	0.02549	6.0
C(2)∙∙∙C(l2′)	0.00457	0.01389	3.1	0.00464	0.01418	3.2
C(5)∙∙∙C(5′)	0.00404	0.01321	2.9	0.00427	0.01383	3.0
H(152)∙∙∙H(5′)	0.00591	0.02296	4.8	0.00587	0.02224	4.7

^(a)^ *E*_int_ was evaluated using Equation (1); ^(b)^ atomic numbering is borrowed from cif file.

## Data Availability

The data supporting this article has been included as part of the ESI. Crystallographic data were deposited with CSD under the numbers 2480902 and 2481076. I/O files are available from the respective author upon reasonable request.

## Supplementary Materials

The following supporting information can be downloaded at: https://www.mdpi.com/article/10.3390/molecules31061047/s1, Computation of the molecular electrostatic potential surface for 2,6-dibromophenol and 2,6-dichlorophenol molecules; Details of periodic (solid-state) DFT calculations; Figure S1: Asymmetric unit in the structure of [TMG•2,6-dichlorophenol] (1:1). Thermal ellipsoids are shown at 50% probability level. Hydrogen bonds are drawn as dashed lines; Figure S2: Asymmetric unit in the structure of [TMG•2,6-dibromophenol] (1:2). Thermal ellipsoids are shown at 50% probability level. Hydrogen bonds are drawn as dashed lines; Figure S3: The molecular electrostatic potential surface (ESP) at 0.001 a.u. for 2,6-dibromophenol (a) and 2,6-dichlorophenol molecules (b). ESP minima and maxima (V_S_ in kcal/mol) are shown as red and green dots respectively; Figure S4: 2D fingerprint plots for the TMG molecule in [TMG•2,6-dichlorophenol] (1:1) and [TMG•2,6-dibromophenol] (1:2); Table S1: Crystallographic data, details of the SC-XRD experiment and structure refinement for two component crystals of glycine betaine with 2,6-dichlorophenol and 2,6-dibromophenol obtained in this study; Table S2: The electron density *ρ*_b_ and the Laplacian of the electron density ∇^2^*ρ*_b_ at the bond critical point and the energy *E*_int_ of the strongest H-bonds and non-covalent interactions of different types in [TMG•2,6-dibromophenol] (1:2); Table S3: Relative contributions of hydrogen bonds and non-covalent interactions to the total energy of intermolecular interactions in crystalline [TMG•2,6-dichlorophenol] (1:1) obtained using Bader analysis of the periodic electron density and two-dimensional fingerprint diagrams; Table S4: Relative contributions of hydrogen bonds and non-covalent interactions to the total energy of intermolecular interactions in crystalline [TMG•2,6-dibromophenol] (1:2) obtained using Bader analysis of the periodic electron density and two-dimensional fingerprint diagrams; Table S5: The electron density *ρ*_b_, the Laplacian of the electron density ∇^2^*ρ*_b_, and the local electronic kinetic energy density *G_b_* at the bond critical point of the C∙∙∙H and H∙∙∙H contacts found from Bader analysis of the crystalline electron density calculated using periodic DFT with PBE-D3 for the naphthalene crystal. The energy of the contacts are given in the last column; Table S6: The electron density *ρ*_b_, the Laplacian of the electron density ∇^2^*ρ*_b_, and the local electronic kinetic energy density *G_b_* at the bond critical point of the C∙∙∙H and H∙∙∙H contacts found from Bader analysis of the crystalline electron density calculated using periodic DFT with PBE-D3 for the anthracene crystal. The energy of the contacts are given in the last column; Table S7: Relative contributions of hydrogen bonds and non-covalent interactions to the total energy of intermolecular interactions in crystalline cytosine obtained using Bader analysis of the periodic electron density and two-dimensional fingerprint diagrams; Table S8: Relative contributions of hydrogen bonds and non-covalent interactions to the total energy of intermolecular interactions in crystalline 3-deazauracil and N-formylglycine obtained using Bader analysis of the periodic electron density and two-dimensional fingerprint diagrams, see [86,87,88,89].
