# Supplementary material for: Tuning Supramolecular Structure in Trimethylglycine Cocrystals: Competition Between Hydrogen and Halogen Bonding upon Cl/Br Substitution
Source: Molecules. 2026 Mar 21;31(6):1047. doi: 10.3390/molecules31061047 (PMC13028978; doi:10.3390/molecules31061047)
Supplement: Supplementary file 1 [file molecules-31-01047-s001.zip › molecules-4177226-supplementary.pdf]

Supplementary Materials

Tuning Supramolecular Structure in Trimethylglycine Cocrystals: Competition Between Hydrogen and Halogen Bonding upon Cl/Br Substitution

Andrei V. Churakov [1], Alexander G. Medvedev [1], Anastasia V. Shishkina [2], Nikita E. Frolov [3] and Mikhail V. Vener [1,*]

[1] N. S. Kurnakov Institute of General and Inorganic Chemistry, Russian Academy of Sciences, Leninskii Prosp. 31, 119991 Moscow, Russia; churakov@igic.ras.ru (A.V.C.); medvedev.chem@gmail.com (A.G.M.)
[2] Scientific Department, Northern (Arctic) Federal University, Severnaya Dvina Emb. 17, 163001 Arkhangelsk, Russia; shishkina.av@gmail.com
[3] V. M. Gorbatov Federal Research Center for Food Systems, Talalikhina St. 26, 109316 Moscow, Russia; frolovne24@gmail.com
* Correspondence: vener@igic.ras.ru or mikhail.vener@gmail.com

Table S1. Crystallographic data, details of the SC-XRD experiment and structure refinement for two component crystals of glycine betaine with 2,6-dichlorophenol and 2,6-dibromophenol obtained in this study.

Figure S1. Asymmetric unit in the structure of [TMG•2,6-dichlorophenol] (1:1). Thermal ellipsoids are shown at 50% probability level. Hydrogen bonds are drawn as dashed lines. .

Figure S2. Asymmetric unit in the structure of [TMG•2,6-dibromophenol] (1:2). Thermal ellipsoids are shown at 50% probability level. Hydrogen bonds are drawn as dashed lines.

Computation of the molecular electrostatic potential surface for 2,6-dibromophenol and 2,6-dichlorophenol molecules.

Figure S3. The molecular electrostatic potential surface (ESP) at 0.001 a.u. for 2,6-dibromophenol (a) and 2,6-dichlorophenol molecules (b). ESP minima and maxima (V_S in kcal/mol) are shown as red and green dots respectively.

Details of periodic (solid-state) DFT calculations.

Table S2. The electron density ρ_b and the Laplacian of the electron density $\nabla^2\rho_b$ at the bond critical point and the energy E_{int} of the strongest H-bonds and non-covalent interactions of different types in [TMG•2,6-dibromophenol] (1:2).

Figure S4. 2D fingerprint plots for the TMG molecule in [TMG•2,6-dichlorophenol] (1:1) and [TMG•2,6-dibromophenol] (1:2).

Table S3. Relative contributions of hydrogen bonds and non-covalent interactions to the total energy of intermolecular interactions in crystalline [TMG•2,6-dichlorophenol] (1:1) obtained using Bader analysis of the periodic electron density and two-dimensional fingerprint diagrams.

Table S4. Relative contributions of hydrogen bonds and non-covalent interactions to the total energy of intermolecular interactions in crystalline [TMG•2,6-dibromophenol] (1:2) obtained using Bader analysis of the periodic electron density and two-dimensional fingerprint diagrams.

Table S5. The electron density ρ_b, the Laplacian of the electron density $\nabla^2\rho_b$, and the local electronic kinetic energy density G_b at the bond critical point of the C···H and H···H contacts found from Bader analysis of the crystalline electron density calculated using periodic DFT with PBE-D3 for the naphthalene crystal. The energy of the contacts are given in the last column.

Table S6. The electron density ρ_b, the Laplacian of the electron density $\nabla^2\rho_b$, and the local electronic kinetic energy density G_b at the bond critical point of the C···H and H···H contacts found from Bader analysis of the crystalline electron density calculated using periodic DFT with PBE-D3 for the anthracene crystal. The energy of the contacts are given in the last column.

Table S7. Relative contributions of hydrogen bonds and non-covalent interactions to the total energy of intermolecular interactions in crystalline cytosine obtained using Bader analysis of the periodic electron density and two-dimensional fingerprint diagrams.

Table S8. Relative contributions of hydrogen bonds and non-covalent interactions to the total energy of intermolecular interactions in crystalline 3-deazauracil and N-formylglycine obtained using Bader analysis of the periodic electron density and two-dimensional fingerprint diagrams.

Table S1. Crystallographic data, details of the SC-XRD experiment and structure refinement for two crystals of trimethylglycine with 2,6-dichlorophenol and 2,6-dibromophenol obtained in this study.

	[TMG•2,6-dichlorophenol] (1:1)	[TMG•2,6-dibromophenol] (1:2)
Formula	$C_{11}H_{15}Cl_2NO_3$	$C_{17}H_{19}Br_4NO_4$
*F*w	280.14	620.97
colour, habit	prism, colourless	prism, colourless
cryst size (mm)	0.20×0.15×0.05	0.20×0.20×0.15
temperature (K)	150	100
crystal system	triclinic	Monoclinic
space group	*P*-1	$P2_1/c$
a (Å)	8.2046(2)	9.1769(6)
b (Å)	8.2258(2)	27.7984(18)
c (Å)	10.3184(3)	8.2098(5)
α (deg)	67.2075(10)	90
β (deg)	87.3091(10)	92.415(2)
γ (deg)	83.0424(10)	90
V ($Å^3$)	637.27(3)	2092.5(2)
Z	2	4
D_c ($g·cm^{-3}$)	1.460	1.971
μ (mm^{-1})	4.571(Cu-Kα)	7.715 (Mo- Kα)
F(000)	292	1200
θ range (deg)	4.65 to 70.00	2.221 to 25.242
refl collcd	25115	31694
indep reflns / R_{int}	2386 / 0.0457	5043 / 0.0621
reflns $I>2σ(I)$	2247	4374
No of param	215	312
GooF on F^2	1.041	1.039
R_1 ($I>2σ(I)$)	0.0263	0.0248
wR_2(all data)	0.0671	0.0556
largest diff peak / hole ($e·Å^{-3}$)	0.31 / -0.21	0.48 / -0.54
CCDC number	2480902	2481076

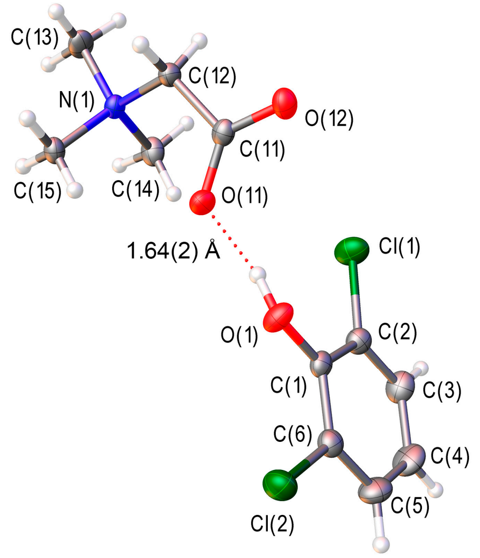

Figure S1. Asymmetric unit in the structure of [TMG•2,6-dichlorophenol] (1:1). Thermal ellipsoids are shown at 50% probability level. Hydrogen bonds are drawn as dashed lines.

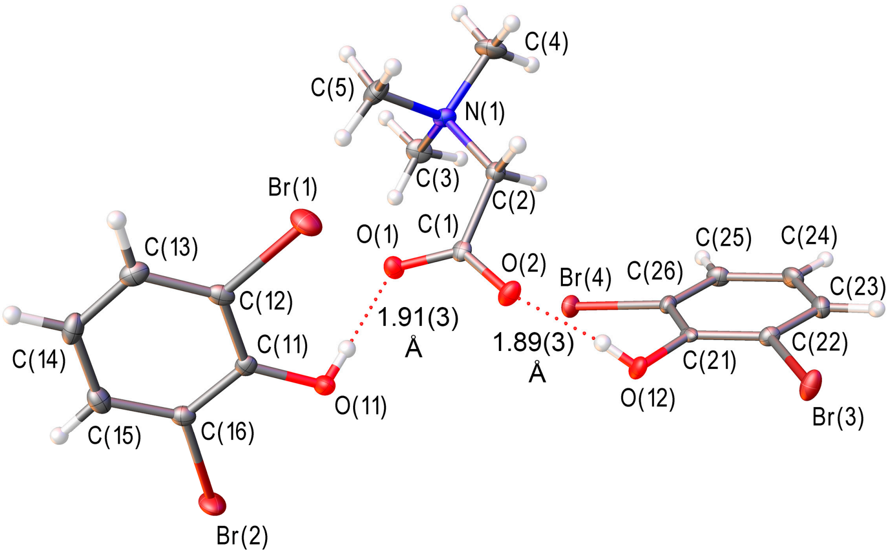

Figure S2. Asymmetric unit in the structure of [TMG•2,6-dibromophenol] (1:2). Thermal ellipsoids are shown at 50% probability level. Hydrogen bonds are drawn as dashed lines.

Computation of the molecular electrostatic potential surface for 2,6-dibromophenol and 2,6-dichlorophenol molecules.

Non-periodic DFT calculations were performed with the Gaussian 09[86] package at the B3LYP level[75,76]. For C,O,H atoms and halogen atoms, the 6-311++g(d,p) and def2-TZVPP basis[87] sets were applied, respectively. The structures of halogenated phenols were fully optimized. The nature of all the stationary points on the potential energy surfaces was confirmed by vibrational analysis. Molecular electrostatic potential surfaces were generated by Gaussian 09, further analyzed by the Multiwfn package[88] and visualized with VMD 2.0 package.[89]

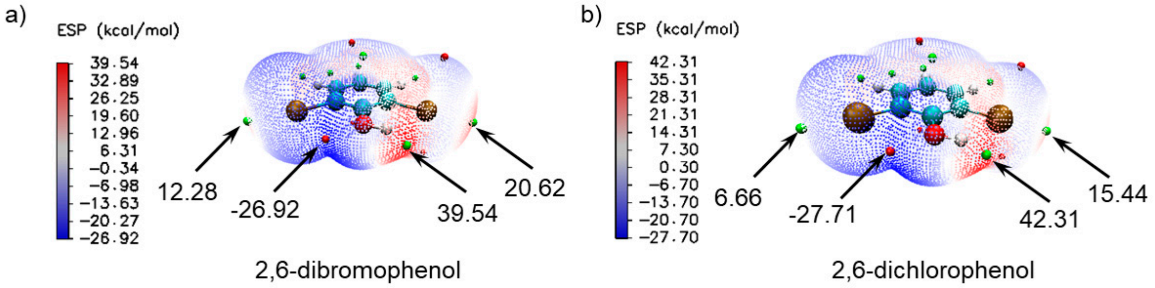

Figure S3. The molecular electrostatic potential surface (ESP) at 0.001 a.u. for 2,6-dibromophenol (a) and 2,6-dichlorophenol molecules (b). ESP minima and maxima (V_S in kcal/mol) are shown as red and green dots respectively.

Details of periodic (solid-state) DFT calculations.

The CRYSTAL parameters describing the level of accuracy in evaluating the Coulomb and Hartree–Fock exchange series were set to 7 7 7 7 15. Tolerances on energies which control the self-consistent field convergence for geometry optimizations and frequency computations were set to 1×10^{-10} and 1×10^{-11} Hartree, respectively. The shrinking factor of the reciprocal space net was set to 3.

The space group and the unit cell parameter of the considered crystal obtained from the X-ray diffraction experiment were fixed, and the structural relaxations were restricted to the positional parameters of the atoms (AtomOnly parameter in Crystal17). The analysis of normal vibrations does not reveal imaginary frequencies for crystals under consideration.

Table S2. The electron density ρ_b and the Laplacian of the electron density $\nabla^2\rho_b$ at the bond critical point and the energy E_{int}[a)] of the strongest H-bonds and non-covalent interactions of different types in [TMG•2,6-dibromophenol] (1:2).

Fragment[b)]	B3LYP [c)]			PBE-D3 [d)]		
	ρ_b (a.u.)	$\nabla^2\rho_b$ (a.u.)	E_{int} (kJ/mol)	ρ_b (a.u.)	$\nabla^2\rho_b$ (a.u.)	E_{int} (kJ/mol)
O(11)–H···O(1)	0.05622	0.14785	46.1	0.06419	0.14683	50.3
O(12)–H···O(2)	0.04564	0.14344	40.0	0.05020	0.14563	42.2
C(2)–H···O(2)	0.01111	0.03371	9.0	0.01121	0.03338	8.9
Br(4)···O(1)	0.01054	0.04015	9.7	0.01104	0.04073	9.8
Br(4)···H(22)	0.00682	0.02212	5.0	0.00695	0.02191	5.0
Br(2)···C(21)	0.00677	0.02017	4.6	0.00662	0.02138	4.8
Br(2)···Br(4)	0.00569	0.01477	3.5	0.00578	0.01395	3.5
C(15)···H(25)	0.00987	0.02890	7.0	0.00671	0.02519	5.6
H(32)···H(32'')	0.00587	0.0225	4.7	0.00563	0.02299	4.8

a) E_{int} was evaluated using Eq. (1);

b) atomic numbering is borrowed from cif file;

c) found from Bader's analysis of the periodic electron density calculated using B3LYP/6-31G**;

d) found from Bader's analysis of the periodic electron density calculated using PBE-D3/6-31G**.

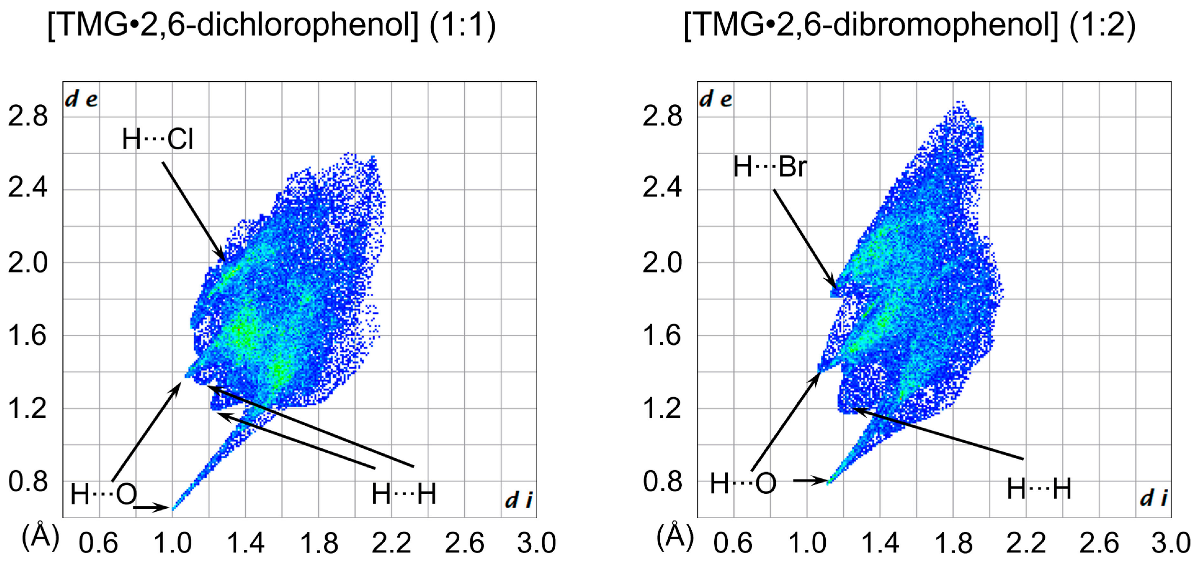

Figure S4. 2D fingerprint plots for the TMG molecule in [TMG•2,6-dichlorophenol] (1:1) and [TMG•2,6-dibromophenol] (1:2).

Table S3. Relative contributions of hydrogen bonds or non-covalent interactions to the total energy of intermolecular interactions in crystalline [TMG•2,6-dichlorophenol] (1:1) obtained using Bader analysis of the periodic electron density (Bader) and two-dimensional fingerprint diagrams (2D-diagram).

Contact	Contribution in %	
	Bader[a)]	2D-diagram
H···O	73.9	37.2
H···Cl	7.8	9.9
H···H	5.5	42.1
H···C	1.1	9.4
Cl···O	3.1	0.6
Other	8.6	0.7

[a)] periodic DFT computations using the B3LYP level.

Table S4. Relative contributions of hydrogen bonds or non-covalent interactions to the total energy of intermolecular interactions in crystalline [TMG•2,6-dibromophenol] (1:2) obtained using Bader analysis of the periodic electron density (Bader) and two-dimensional fingerprint diagrams (2D-diagram).

Contact	Contribution in %	
	Bader [a)]	2D-diagram
H···O	57.4	40,1
H···Br	14.0	27.6
H···H	4.3	28.1
H···C	9.1	2.4
Br···O	8.4	1.3
Other	6.8	0.5

[a)] periodic DFT computations using the B3LYP level.

Table S5. The electron density ρ_b, the Laplacian of the electron density $\nabla^2\rho_b$, and the local electronic kinetic energy density G_b at the bond critical point of the C···H and H···H contacts found from Bader analysis of the crystalline electron density calculated using periodic DFT with PBE-D3 for the naphthalene crystal. The energy E_{int} [a)] of the contacts are given in the last column.

Contact [b)]	ρ_b (a.u.)	$\nabla^2\rho_b$ (a.u.)	G_b (a.u.)	E_{int} (kJ mol^{-1})
C(5)···H(1’)	0.00448	0.01423	0.00272	3.06
C(2’)···H(5)	0.00302	0.00909	0.00177	2.00
C(4’)···H(2)	0.00421	0.01396	0.00263	2.96
H(2’)···H(1)	0.00571	0.02000	0.00378	4.26
H(1)···H(1’)	0.00419	0.01545	0.00285	3.21
H(1)···H(5)	0.00403	0.01402	0.00260	2.93
H(4)···H(4’)	0.00389	0.01346	0.00252	2.84

[a)] E_{int} was evaluated using Eq. (1);

[b)] atomic numbering is borrowed from NAPHTA33.cif file.

Table S6. The electron density ρ_b, the Laplacian of the electron density $\nabla^2\rho_b$, and the local electronic kinetic energy density G_b at the bond critical point of the C···H and H···H contacts found from Bader analysis of the crystalline electron density calculated using periodic DFT with PBE-D3 for the anthracene crystal. The energy E_{int} [a)] of the contacts are given in the last column.

Contact [b)]	ρ_b (a.u.)	$\nabla^2\rho_b$ (a.u.)	G_b (a.u.)	E_{int} (kJ mol^{-1})
C(4)···H(4')	0.00926	0.02795	0.00582	6.59
C(7)···H(1)	0.00499	0.01585	0.00304	3.49
H(2')···H(1)	0.00468	0.1633	0.00302	3.40
H(2)···H(4)	0.004711	0.1621	0.00299	3.36
H(5)···H(1')	0.00455	0.01592	0.00294	3.30
H(1)···H(1')	0.00321	0.01173	0.00219	2.46
H(3)···H(3')	0.00489	0.01694	0.00312	3.50

[a)] E_{int} was evaluated using Eq. (1);

[b)] atomic numbering is borrowed from ANTCEN01.cif file.

Table S7. Relative contributions of hydrogen bonds and non-covalent interactions to the total energy of intermolecular interactions in crystalline cytosine[a)] obtained using Bader analysis of the periodic electron density (Bader) and two-dimensional fingerprint diagrams (2D-diagram).

Contact	Contribution in %	
	Bader [b)]	2D-diagram
H···O	45.3	25.1
H···H	0.0	32.6
H···N	34.1	19.1
H···C	8.7	10.2
N···O	3.1	1.6
N···N	2.6	0.4
N···C	6.1	5.3
C···C	0.0	3.3
C···O	0.0	2.3
O···O	0.0	0.1
Other	-	-

[a)] CSD Ref. code is CYTSIN01;

[b)] periodic DFT computations at the PBE-D3 level.

Table S8. Relative contributions of hydrogen bonds and non-covalent interactions to the total energy of intermolecular interactions in crystalline 3-deazauracil and N-formylglycine obtained using Bader analysis of the periodic electron density [a] (Bader) and two-dimensional fingerprint diagrams (2D-diagram).

Contact	3-deazauracil [b]		N-formylglycine [c]	
	Contribution in %	Contribution in %	Contribution in %	Contribution in %
	Bader	2D-diagram	Bader	2D-diagram
H···O	86.0	35.0	88.0	59.5
O···C	3.1	5.7	6.6	3.9
H···H	-	30.1	3.0	25.9
H···C	6.1	23.0	-	4.2
H···N	-	3.7	-	1.5
N···O	4.8	1.8	-	1.8
Other	-	0.7	2.4	3.2

[a] periodic DFT computations using the PBE-D3 level; [b] CSD Ref. code is CAXKOB12;
[c] CSD Ref. code is EVIQAB.
